# Circadian stabilization loop: the regulatory hub and therapeutic target promoting circadian resilience and physiological health

**DOI:** 10.12688/f1000research.126364.2

**Published:** 2022-12-12

**Authors:** Eunju Kim, Seung-Hee Yoo, Zheng Chen

**Affiliations:** 1 Department of Biochemistry and Molecular Biology, McGovern Medical School, The University of Texas Health Science Center at Houston (UTHealth Houston), Houston, TX, 77030, USA

**Keywords:** Circadian oscillator, core loop and stabilization/secondary loop, REV-ERBs and RORs, ligands and drugs, circadian amplitude and resilience, physiological health, healthy aging

## Abstract

The circadian clock is a fundamental biological mechanism that orchestrates essential cellular and physiological processes to optimize fitness and health. The basic functional unit is the cell-autonomous oscillator, consisting of intersecting negative feedback loops. Whereas the core loop is primarily responsible for rhythm generation, auxiliary loops, most notably the secondary or stabilization loop, play pivotal roles to confer temporal precision and molecular robustness. The stabilization loop contains opposing nuclear receptor subfamilies REV-ERBs and retinoic acid receptor-related orphan receptors (RORs), competing to modulate rhythmic expression of the basic helix-loop-helix ARNT like 1 ( *Bmal1*) genes in the core loop as well as other clock-controlled genes. Therefore, REV-ERBs and RORs are strategically located to interface the oscillator and the global transcriptomic network, promoting cellular homeostasis and physiological fitness throughout lifespan. Disruption of REV-ERB and ROR functions has been linked with diseases and aging, and pharmacological manipulation of these factors has shown promise in various mouse disease models. Nobiletin is a natural compound that directly binds to and activates RORα/γ, modulating circadian rhythms, and shows robust *in vivo* efficacies to combat clock-associated pathophysiologies and age-related decline. Results from several studies demonstrate an inverse relation between nobiletin efficacy and clock functional state, where nobiletin elicits little effect in young and healthy mice with growing efficacy as the clock is perturbed by environmental and genetic challenges. This mode of action is consistent with the function of the stabilization loop to promote circadian and physiological resilience. Future studies should further investigate the function and mechanism of REV-ERBs and RORs, and test strategies targeting these factors against disease and aging.

## The circadian timing system and health implications

Circadian rhythms are daily cycles of intrinsic processes in living organisms. While light/dark cycles of our environment are the predominant input (or zeitgeber, time giver) to reset our internal rhythms, it is now clear that other factors including feeding-fasting state, nutrients, physical activity, and temperature are all capable of manipulating the circadian cycle. ^1^^–^^3^ Fundamentally, the circadian timing system is a molecular circuit governing cellular and physiological homeostasis throughout lifespan. Alterations to this clock machinery, by either environmental stresses or genetic defects, have been shown to cause or correlate with dysfunction of diverse physiological processes and increased risks for various diseases involving both peripheral organs and the brain. ^4^^–^^6^

At the pinnacle of the circadian timing system is the master pacemaker located in the suprachiasmatic nucleus (SCN) of the hypothalamus. The SCN clock synchronizes semi-autonomous cellular oscillators in other brain regions and peripheral organs through neuronal and hormonal signals. ^7^^–^^9^ The ubiquitous cellular oscillator, present in the SCN and throughout the body, contains interlocked transcriptional and translational feedback loops controlling the expression of downstream target genes. ^10^ The core clock genes functioning in the oscillator include circadian locomotor output cycles kaput ( *Clock*)/neuronal PAS domain-containing protein 2 ( *Npas2*), basic helix-loop-helix ARNT like 1 ( *Bmal1*), period 1 ( *Per1*)/period 2 ( *Per2*)/period 3 *(Per3*), cryptochrome 1 ( *Cry1*)/cryptochrome 2 ( *Cry2*), *Rev-erba*/ *Rev-erbb* (nuclear receptor subfamily 1 group D member 1/2 ( *Nr1d1*) */2*)), and retinoic acid receptor-related orphan receptor alpha *(Rora*)/retinoic acid receptor-related orphan receptor beta ( *Rorb*)/Retinoic acid receptor-related orphan receptor gamma ( *Rorc*) ( *Nr1f1/2/3*), D-box binding protein ( *Dbp*), and nuclear factor, interleukin-3 regulated protein ( *Nfil3*). By acting on consensus promoter elements or directing the expression of secondary regulators of gene expression, the encoded core clock proteins play a prevalent role in the global gene expression landscape where more than 80% of genes have been shown to oscillate in at least one location in the body. ^11^^,^^12^

Perhaps one of the most important physiological functions of the clock is to safeguard energy homeostasis. ^13^^,^^14^ It has been postulated that an evolutionary origin of the circadian system is energy partitioning: photosynthesis using oxygen during the day and anaerobic metabolism including nitrogen fixation at night. ^15^ In mammals, central and peripheral clocks coordinately drive rhythmic expressions of metabolic-related genes in organs with high metabolic activity including liver, muscle, and adipose tissue. ^3^^,^^16^^–^^18^ Over the past 15 years or so, a growing body of evidence has established that the clock gene machinery influences energy homeostasis directly and genetic mutations in clock genes lead to metabolic dysfunctions, including deficient insulin resistance, glucose intolerance, leptin resistance, and abnormal glucocorticoid and melatonin levels. ^17^^,^^19^^,^^20^ In accordance, human subjects who were exposed to a controlled circadian misalignment condition displayed glucose intolerance, insulin resistance, and other comorbidities. ^21^^–^^23^ In addition, our lifestyle choices that affect circadian rhythms may also evoke adverse metabolic consequences. For example, external stimuli including abnormal light exposure, ^24^ jet-lag, ^20^^,^^25^ and high-fat diet induced-obesity ^26^^,^^27^ can trigger desynchronization of the internal clock accompanied by many tissue disorders. Furthermore, sleep deprivation, a common occurrence in modern lifestyle, is associated with increased body mass index and type 2 diabetes incidence and has been identified as an independent risk factor for hypertension, obesity, and coronary heart disease. ^28^^,^^29^ In addition, sleep and feeding alterations and shift work are highly correlated with elevated metabolic syndrome markers such as triglycerides, and lower high-density lipoprotein (HDL)-cholesterol levels. ^29^^–^^31^

Dysregulated clocks are also involved in brain dysfunction and diseases. ^32^^–^^34^ Sleep is well known to be regulated by the clock, and elegant studies combining human genetics and mechanistic investigation have revealed molecular links between several mutations in clock genes, including *PER2* and casein kinase I isoform delta ( *CSNK1D*), and sleep disorders. ^35^ An emerging area of interest is the crosstalk between the clock and neurodegenerative diseases. ^36^^–^^38^ Circadian clocks have been shown to control several aspects of brain functions linked to neurodegeneration including dopamine synthesis, inflammatory response, oxidative stress, and cellular metabolism. ^33^^,^^39^ Consistently, circadian and sleep disruptions are closely associated with neurodegenerative diseases including Alzheimer’s disease and Parkinson’s disease, ^40^ as evidenced by amyloid-beta (Aβ) oscillation, ^41^ sundowning behaviors, ^42^ and neuronal inflammation in mouse genetic mutants. ^40^

Given the fundamental role of the clock in cellular and physiological homeostasis and the myriads of chronic diseases associated with circadian dysregulation, it is not surprising that age-related decline over time is strongly correlated with and likely exacerbated by dysfunction in the clock system. ^43^ It is well known that a number of physiological parameters display blunted circadian rhythms during aging, including sleep, temperature, and hormone secretion. ^43^^,^^44^ More recently, global transcriptomic profiling revealed profound rewiring in the clock network, notably dampening of oscillatory gene expression in accordance with the physiological decline. ^45^ A key role of the circadian rhythms in aging is further highlighted by two large-scale gene profiling studies where circadian gene expression changes emerged from unbiased analyses as a top underlying pathway during aging. ^46^^,^^47^ For example, a comparative multi-tissue gene profiling approach was undertaken to search for pathways correlated with maximum lifespan in 26 species, and identified the circadian system as a pillar that governs metabolic and inflammatory pathways for longevity regulation. ^47^ Furthermore, interventional fasting paradigms designed to incorporate circadian timing were recently reported to markedly prolong lifespan in *Drosophila* and mice, ^48^^,^^49^ including 35% lifespan extension in male mice. The convergent spotlight on circadian remodeling during aging provides compelling evidence for the notion that a robust circadian system is key to health and healthspan. ^50^

## The stabilization loop

**Figure 1.**
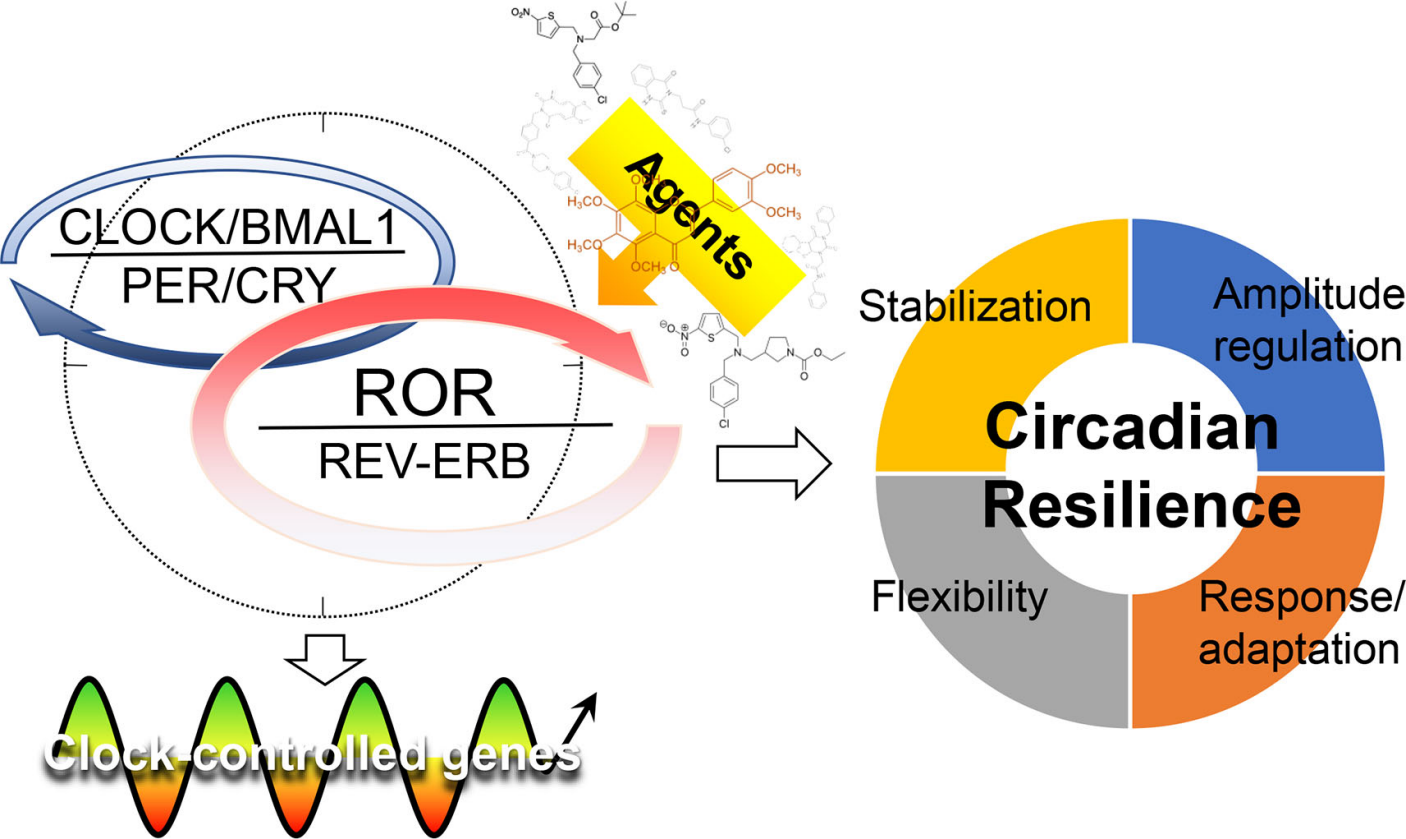
The stabilization loop of the circadian oscillator is strategically situated at the interface of the rhythm-generating core loop and the circadian output network. The core oscillator mainly consists of core and stabilization loops as indicated by the dotted circle. The REV-ERB and retinoic acid receptor-related orphan receptors (RORs) compete at the consensus ROR response elements (ROREs) of target gene promoters, including basic helix-loop-helix ARNT like 1 ( *Bmal1*) and many clock-controlled genes (CCGs), to regulate circadian transcription in a tissue-specific manner. They may interact *via* other mechanisms and in clock-independent processes – see text for details. REV-ERBs and RORs play regulatory roles in many tissue and organismal functions and targeting these receptors by small-molecule agents may strengthen circadian resilience, ultimately conferring beneficial effects to promote health and health span. CLOCK, circadian locomotor output cycles kaput; CRY, cryptochrome; PER, period.

The core loop of the oscillator is primarily responsible for generating the near-24hr rhythm *via* the negative feedback between CLOCK/BMAL1 and CRY/PER. Through binding to E-box elements, the CLOCK/BMAL1 heterodimer activates the expression of many Clock-Controlled Genes (CCGs). As PER/CRY proteins accumulate and reach critical levels in the cytoplasm, they translocate to the nucleus to inhibit the activity of CLOCK/BMAL1, thereby inhibiting their own transcription. ^10^ On the other hand, the secondary loop, mainly involving the opposing transcription factors REV-ERBs and RORs, ^51^^,^^52^ confers stability and robustness for the core loop, and is also strategically located at the interface between the core oscillator and many downstream clock-controlled genes ( Figure 1). Growing evidence suggests a regulatory and therapeutic potential of the stabilization loop in physiology, disease, and aging. ^53^

REV-ERBs and RORs, the main components of the stabilization loop, are multi-functional nuclear receptors to repress and activate target gene expression, respectively. ^52^^,^^54^^,^^55^ REV-ERBs and RORs bind as monomers to the same consensus sequence, namely the ROR response elements (ROREs, or RREs) consisting of an (A/G) GGTCA flanked by an A/T-rich 5’ in the regulatory regions of the target genes. ^56^^–^^58^ In the core oscillator, perhaps the most important function of REV-ERB/ROR is to maintain and fine-tune the oscillatory expression of *Bmal1*, encoding the chief circadian transcription activator in the core loop, thereby serving as a stabilizing mechanism. ^52^^,^^55^^,^^59^ In addition, several other core clock genes have been shown to possess the RORE elements in their promoter region, ^60^ suggesting an important stabilizing function by these opposing regulators. More broadly, gene expression and profiling studies have illustrated a prominent role of REV-ERBs and RORs in transcriptomic landscape in various tissues/cells. ^61^^–^^65^ For example, studies of liver-specific RORα/RORγ double knockout mice revealed a key role of RORs in lipogenesis, *via* direct transcriptional regulation of the insulin-induced gene 2 ( *Insig2*)-sterol regulatory element-binding protein 1 ( *SREBP*) cascade. ^65^

Traditionally REV-ERBs and RORs are considered to interact and compete as repressors and activators on shared target genes. While *Rorα* expressions display relatively moderate circadian oscillatory amplitude, *Rorγ* is more rhythmic, exhibiting a strong oscillatory pattern of expression in several tissues such as liver, brown adipose tissue (BAT), kidney, and small intestines with peak expression around ZT16, but not in the hypothalamus, muscle, or brainstem. ^66^^–^^68^ Notably, *Rev-erbs* are among the most oscillatory genes (highest amplitude) in both protein and mRNA expression. ^64^^,^^69^ It is believed that their opposing functions and circadian patterns (expression and promoter recruitment) play a key role in amplitude regulation of clock-controlled genes. In a pioneering study, the mRNA oscillation of *Bmal1, Clock*, and *Cry1* genes show strong amplitude in wild-type animals but is diminished in *Rev-erbα* deficient mice. ^55^ Although dispensable for *Bmal1* oscillation per se, RORs were found to contribute to its amplitude. ^52^^,^^70^^,^^71^ More recently, correlation analysis of publicly available mouse circadian transcriptomic datasets, including both microarray and RNA-sequencing, identified a strong correlation between *Rorc* expression level and amplitude with the percentage of cycling transcripts in respective tissues, consistent with a regulatory role of RORγ in circadian oscillation. ^72^ In addition to jointly regulating *Bmal1* transcription, additional modes of genetic and molecular interplay exist between REV-ERBs and RORs. *Rorc* itself contains a functional RORE element in its promoter, therefore subject to transcriptional regulation by REV-ERBs/RORs. ^60^ Moreover, molecular studies have demonstrated a facilitated recruitment mechanism where REV-ERBs are recruited to the target gene promoter by RORs, in a process that requires chromatin remodeling by SWI/SNF factors. ^73^ These observations suggest an interconnectivity, rather than a simple competition, relationship between these master regulators. As discussed below, our studies of an ROR agonist, nobiletin (NOB), provide further evidence that RORs, and likely REV-ERBs, regulate circadian gene expression levels and amplitude in a context-dependent manner, potentially dictated by an inherent requirement to maintain circadian and physiological resilience.

Consistent with the broad gene regulatory roles of REV-ERBs and RORs, mouse genetic mutants exhibit various circadian and physiological phenotypes. *Rev-erba* ( *Nr1d1*)-deficient mice showed disrupted circadian rhythms including a shorter period length (0.5 h) and exaggerated light-induced phase shifts compared to wild-type (WT) mice. ^55^^,^^74^ *Rev-erbb* ( *Nr1d2*) knockout (KO) mice also displayed a strong diurnal change of gene expression including inhibition of *Bmal1* transcription. ^51^ While individual KO mice retained circadian rhythmicity, *Nr1d1/2* double knockout led to severe disruption of overt rhythms, consistent with functional redundancy between these two subtypes. ^70^^,^^75^^,^^76^ *Rora*- and *Rorb*-deficient mice were reported to display altered circadian behavior such as circadian locomotor activity and shortened period length, while no significant alteration in wheel activity is apparent in *Rorc*-deficient mice. ^52^^,^^70^^,^^71^^,^^77^^,^^78^ These results indicated that REV-ERBs and RORs are required for the maintenance of normal circadian behavior and period length.

With respect to tissue physiology, REV-ERBs and RORs show overlapping but distinct expression patterns and their deficiency led to a wide range of other physiological deficits. REV-ERBα and REV-ERBβ are expressed in skeletal muscle, adipose tissue, liver, and brain with tissue-specific patterns. Whereas REV-ERBα is broadly expressed in a rhythmic manner in many tissue types with robust amplitude, REV-ERBβ is highly expressed in fewer tissues including certain brain regions (pineal and prefrontal cortex), thyroid, uterus, and pituitary. Deficiency of both *Rev-erbs* causes liver steatosis, in contrast to relatively minor changes upon loss of each subtype alone. ^75^^,^^79^

Like REV-ERBs, the three members of the ROR subfamily, RORα, RORβ, and RORγ, display significant sequence similarities. RORα is expressed broadly, notably in skeletal muscle, liver, kidney, lungs, adipose tissue, skin, and brain. ^61^ *Rora* KO ( *Rora* ^−/−^) and *staggerer* mutant ( *Rora* ^*sg/sg*^) mice displayed debilitating cerebellar ataxia and are mostly infertile. ^52^^,^^71^^,^^80^^,^^81^ RORα-deficient mice also showed a multitude of other defects including thin long bones ^82^ and abnormal retinal development, ^83^^,^^84^ the latter corresponding to high expression levels of RORα in the ganglion cell layer, the inner nuclear layer, and cone photoreceptors in the outer layer. RORβ expression is more limited, mainly in the nerve system. *Rorb* ^−/−^ mice exhibited reproductive abnormality and serious degeneration of postnatal retina. ^59^ RORγ is expressed in several peripheral tissues including skeletal muscle, liver, kidney, adipose tissue, and particularly thymus. ^61^ In accordance, RORγ KO led to reduced levels of thymocytes and abnormal lymphoid organ development. ^85^ This and many other studies have since established a key role of RORyt as the master transcription factor for Th17 cell development, although circadian clock involvement in this function is not fully understood. Several studies have also examined double disruption of RORs, providing evidence for their overlapping functions. As mentioned above, in the liver where both RORα and RORγ are expressed, double *Rora/c* KO led to strong disruption of lipogenesis *via* a direct regulation of the *Insig2* gene. ^65^

## Therapeutic relevance of REV-ERBs and RORs

REV-ERBα and RORs have been implicated in various diseases including metabolic diseases, immune diseases, and cancers. ^53^^,^^61^^,^^86^^,^^87^ REV-ERBα and RORs show altered expressions and disrupted rhythms during disease development. ^88^^–^^90^ Furthermore, alteration of REV-ERBα and RORs affects the organism susceptibility to diseases in both humans and mice and is involved in many pathways associated with pathological processes and diseases. ^61^^,^^87^^,^^90^^,^^91^^–^^93^

### Metabolic disorders

Myriad studies have illustrated a regulatory role of REV-ERBs and RORs in energy metabolism. REV-ERBα was found to regulate *de novo* glucose synthesis. ^94^^–^^96^ In accordance, REV-ERBα-deficient mice showed a higher plasma glucose level, ^75^^,^^97^ whereas activation of REV-ERBα diminished plasma glucose levels, improving disease phenotypes. ^94^^–^^97^ RORs are also involved in glucose metabolism. Single nucleotide polymorphism in *RORA* (rs7164773) has been shown to correlate with increased risk of type 2 diabetes in the Mexico Mestizo population, providing human genetic evidence for a role of RORα in insulin sensitivity. ^98^ In addition, it was reported that RORα was required for the secretion of FGF21, a hormone associated with glucose tolerance and hepatic lipid metabolism. ^99^^–^^101^ In another study, RORγ was found to regulate transcription of various genes involved in glucose metabolism including glucose-6-phosphatase ( *G6p*), phosphoenolpyruvate carboxykinase 1 ( *Pck*) *,* and glucose transporter 2 ( *Glut2*), and *Rorc*-deficient mice in fact displayed a significantly higher insulin sensitivity and glucose tolerance than WT mice, particularly at ZT (zeitgeber time, with ZT0 and ZT12 corresponding to light on and off respectively) 4–6. ^102^ In pharmacological studies, SR1078, an agonist of RORα and RORγ, was able to improve insulin sensitivity, blood glucose, and triglyceride levels in diabetic rodents. ^103^ In comparison, mice treated with SR3335, an RORα inverse agonist, showed dramatically decreased glucose levels in the plasma compared with the control mice, by inhibiting *Pck* expression and gluconeogenesis. ^104^

With respect to lipid metabolism, *Rev-erba* ^−/−^ mice showed increased very low-density lipoprotein (VLDL) and triglyceride levels, consistent with the observed upregulation of apolipoprotein C-III ( *Apoc3*), a critical regulator in triglyceride metabolism. ^105^^,^^106^ Depletion of both *Rev-erbs* in the liver synergistically de-repressed several metabolic genes as well as genes that control the positive limb of the molecular clock. ^107^ Consistent with these genetic results, administration of the REV-ERB agonist SR9009 decreased cholesterol levels in the plasma in both wild-type and low-density lipoprotein receptor ( *Ldlr*) null mice through downregulating cholesterol biosynthesis gene expression. ^108^ Extensive mouse studies also point to a key role of RORs in lipid metabolism. In *Rorα* ^*sg/sg*^ *staggerer* mice, expression levels of hepatic sterol regulatory element-binding protein 1, isoform c ( *Srebp-1c*), and fatty acid synthase ( *Fas*) were decreased, whereas expression of PGC-1α and β, coactivators involved in oxidative metabolism and gluconeogenesis, were elevated. ^63^^,^^109^ At the molecular level, gene expression and cistrome analysis showed that RORα and/or RORγ broadly regulate genes involved in lipid metabolism in both liver and muscle tissues. ^65^^,^^102^^,^^110^^,^^111^ Furthermore, structural and biochemical studies identified lipid moieties, mainly cholesterol metabolic intermediates, as possible endogenous ligands for RORα/γ, consistent with the notion that RORs may function as a lipid sensor in the regulation of lipid metabolism. ^63^^,^^109^^,^^110^^,^^112^^–^^115^

### Immune diseases

Mounting evidence indicates circadian rhythms in immunity and inflammation. For example, rheumatoid arthritis patients exhibit diurnal variations in functional disability such as joint pain and stiffness in morning time. ^116^^,^^117^ REV-ERBα deletion abolished the diurnal rhythms of various inflammatory factors and aggravates inflammation in diseases including autoimmune encephalomyelitis, ^118^^,^^119^ fulminant hepatitis, ^91^ neuroinflammation, ^120^^,^^121^ heart failure, ^122^^,^^123^ myocardial infarction, ^124^ and ulcerative colitis. ^118^^,^^125^ At the molecular level, REV-ERBα regulates rhythmic transcription of inflammation-related genes involved in macrophage polarization, immune cell differentiation, and NF-κB signaling. ^90^^,^^125^ For example, REV-ERBα was found to obstruct NF-κB signaling in human endometrial stroma cells and macrophages/microglia cells in mouse models, suppressing expression of inflammatory genes such as *IL-1β*, *IL-6*, *IL-18*, tumor necrosis factor alpha ( *Tnfα*), NACHT, LRR and PYD domains-containing protein 3 ( *Nlrp3*), and C-C motif chemokine 2 ( *Ccl2*). ^90^^,^^120^^,^^121^^,^^126^ Activation of REV-ERBα by SR6472 inhibits NF-κB signaling and NLRP3 inflammasome activity to prevent cytokines and chemokines productions, consistent with an anti-inflammatory role of REV-ERBα. ^2^^,^^90^^,^^121^

RORs also play important roles in immunity. ^87^ Extensive research has established RORγt, a subtype of RORγ, as a master regulator of Th17 cell differentiation and therefore highly involved in autoimmune diseases. ^127^ In Th17 cells, RORγt is expressed at dramatically higher levels during daytime than at nighttime. ^128^ This diurnal expression pattern in turn up-regulates BMAL1-dependent Rev-erb expression during daytime and conversely represses NFIL3 transcription. Given the central role of RORγt in Th17 cells, several compounds targeting RORγt have been tested in autoimmune disease models. For example, SR1001, an RORα and RORγ inverse agonist, inhibited Th17 cell differentiation under autoimmune disease conditions. ^129^ Moreover, this effect is associated with decreased expression of several cytokines such as IL17A, IL17F, IL21, and IL22 by specially targeting TH17. ^130^^,^^131^ Likewise, SR2211, an RORγ inverse agonist, suppressed Th17 cell differentiation and reduced IL17a and IL23R expression levels as well as intracellular IL17 protein level. ^132^

### Brain diseases

Circadian disruption can adversely impact brain development and function, potentially leading to various mood and neurological disorders. ^33^^,^^38^ Previously, *Rev-erbb* knockout mice were found to exhibit enhanced anxiety, and treatment of an REV-ERB agonist showed anxiolytic effects. ^133^ On the other hand, acute administration of SR8278, a REV-ERB antagonist, improves anxiety symptom and maniac-like behavior. ^134^^,^^135^ Furthermore, REV-ERBα was shown to diminish fatty acid-binding protein 7 ( *Fabp7*) expression, thereby impairing neuronal differentiation and depleting neuronal progenitor cells. ^136^^,^^137^ Relatedly, deficiency of REV-ERBα adversely affected hippocampal neurogenesis, which contributes to altered mood behaviors. ^138^

RORα is highly expressed in several brain regions such as cerebellar Purkinje cells (PC) and thalamus, and functions to regulate brain development. ^61^^,^^139^^,^^140^ The classical RORα-deficient *staggerer* mice have been shown to present severe ataxia because of cerebellar neurodegeneration ^80^^,^^81^^,^^141^ and abnormal PC development. ^80^^,^^141^^,^^142^ Likewise, *Rora* KO mice exhibit reduced numbers and sizes of PC in the cerebellar region reminiscent of clinical observations from patients with autism-spectrum disorder (ASD). ^143^^,^^144^ RORα also showed neuroprotective effects in astrocytes and neurons during hypoxia. ^112^^,^^145^ RORβ is highly expressed in the retina, pineal gland, and suprachiasmatic nucleus, and has been implicated in visual function, motor ability, and circadian rhythms. ^61^^,^^78^^,^^146^ For example, RORβ-deficient mice showed abnormal motor and olfactory functions, anxiety control, and alterations in circadian behavior. ^77^ The noteworthy question regarding a potential functional overlap in the neuronal system between RORα and RORβ remains to be investigated.

### Muscle pathologies

REV-ERBs (α and β) and RORs (α and γ) are highly expressed in the skeletal muscle where they modulate myofiber types and energy metabolism ^61^^,^^147^ and may be targeted against myopathies. ^148^ In an early study, REV-ERBα-deficient mice showed a marked increase in the relative amount of the slow (type I) myosin heavy chain (MyHC) isoform compared to WT controls. ^147^ Extensive research since has further revealed the regulatory roles of REV-ERBs in skeletal muscle function. ^86^^,^^149^^,^^150^ For example, REV-ERBβ has been implicated in skeletal muscle lipid metabolism since ectopic expression of its dominant-negative form upregulated expression of genes associated with fatty acid uptake in skeletal muscle. ^151^ Consistently, SR8278, an antagonist of REV-ERBs, was found to activate expression of myogenesis genes including Myogenic determination 1 ( *Myod*), Myogenin ( *Myog*), and Major histocompatibility complex 3 ( *Mhc3*), suggesting a role of REV-ERBs in myogenesis. ^152^

Loss-of-function studies also suggest an important role of RORα in skeletal muscle metabolism. ^153^ For example, ectopic expression of a dominant-negative RORα in C2C12 cells or mouse skeletal muscle broadly alters the expression of genes associated with lipid metabolism, lipogenesis, and energy expenditure, including carnitine palmitoyltransferase-1 (Cpt1), caveolin 3 (Cav3), and Srebp1c and its downstream targets. ^110^^,^^154^

### Cancer

REV-ERBα has been implicated in the progression and development of various cancers. ^155^ Activation of REV-ERBα by SR9009 and SR9011 was found to confer cancer cell-selective cytotoxicity as well as *in vivo* efficacy against glioma, and autophagy and lipogenesis were identified as cellular hallmarks closely associated with this anti-cancer activity. ^156^ In a recent study investigating lung adenocarcinoma-associated cachexia, ^157^ REV-ERBα functions as a key effector whose exaggerated turnover contributes to gluconeogenesis gene induction and glucose production in mice.

A number of studies have shown that RORα expression is significantly decreased during tumor development and progression, and exogenous RORα expression repressed cell proliferation and tumor growth. ^158^^–^^163^ For example, down-regulated RORα expression has been observed in colorectal cancer and mammary cancer, and is associated with poor prognosis in patients with hepatocellular and breast carcinoma. ^158^^,^^159^^,^^161^^,^^162^^,^^164^^,^^165^ Conversely, restoring RORα expression suppressed cell migration and tumor growth of breast cancer cells as well as metastasis in nude mice, which was accompanied by up-regulated expression of semaphorin 3F (SEMA3F), a tumor suppressor that reduces tumor growth and invasion. ^161^ In colon cancer HCT116 cells treated with DNA-damage agents, a p53-RORα crosstalk was required for apoptosis, where *Rora* gene transcription was dependent on p53 and RORα in turn rendered greater p53 protein stability. ^166^ In RORγ deficient mice, there was an aggravated development of T-cell lymphomas within the first months after birth, which rapidly metastasized to other organs including liver and spleen. ^167^

## Nobiletin (NOB): A natural ROR agonist

NOB is a natural bioactive polymethxylated flavonoid. ^168^^,^^169^ Many studies have provided functional evidence both *in vitro* and *in vivo* for its biological efficacy in diverse disease models, ^168^^,^^170^^,^^171^ including metabolic diseases and inflammation. In our previous unbiased chemical screen, we identified NOB, along with its close analog tangeretin, as a clock-enhancing small molecule in cell-based circadian reporter assays. ^172^ Focusing on NOB, we demonstrated a circadian clock-dependent efficacy to blunt obesity and metabolic dysfunction in mouse models, and importantly identified RORα and RORγ as its direct targets *via* radioactive ligand binding assays. NOB shows robust binding to the LBDs of RORα and RORγ, with somewhat higher affinity for RORγ. Currently there is no functional evidence to suggest subtype selectivity analogous to CRY-selective compounds. ^173^ Subsequent published studies, from our group and others, have provided further evidence that NOB plays a beneficial role in strengthening circadian physiologies in various mouse models, including aging, metabolic disorders, cardiovascular disease, and Alzheimer’s disease (AD). ^171^^,^^174^^–^^182^ In further support of NOB as an anti-inflammatory agent, recent studies demonstrated a potent role of NOB against neuroinflammation and astrogliosis, accompanied by mitigation of Aβ plaque deposition, in an amyloid AD mouse model. ^183^ Given the increasing appreciation of circadian rhythms in aging, recent studies have also tested its effect in aging models. In naturally aged mice fed with either regular or high-fat diets (HFD), NOB was found to promote healthy aging at several levels, including metabolic homeostasis, inflammatory markers, tissue functions, and systemic behaviors. ^175^ An important target organ is skeletal muscle, where circadian gene reprogramming and metabolomic alteration support an improved mitochondrial function, accompanied by respiratory supercomplex formation. Notably, while NOB-mediated improvement in general healthy aging parameters seems more pronounced in metabolically challenged aged mice (HFD fed) than in those fed with regular diet, the latter group showed an extension of median lifespan, but not maximum lifespan. ^175^ In comparison, NOB was found to exhibit longevity effects in *C. elegans*, extending median lifespan by up to 21%. ^184^ Overall, these and many other studies underscore a strong health-promoting effect of NOB, at least in part via circadian mechanisms.

Mechanistic studies have begun to shed light on the circadian modulatory action of NOB. In addition to its clock amplitude-enhancing effects, NOB also alters the other two cardinal circadian parameters, period, and phase, at least *in vitro.* ^172^^,^^182^ Following chronic treatment *in vivo* (10-12 weeks), NOB was able to strengthen oscillatory amplitude, as well as peak expression, of core clock components at both transcript and protein levels in HFD-fed mice, and wheel-running activity was also increased at nighttime. ^172^ Given the extensive crosstalk between clocks and metabolism/physiology, these overt enhancements of circadian rhythms may result from both direct and indirect effects of NOB on the core oscillator/RORs and clock-regulated downstream functions, respectively. Acute *in vivo* effects on circadian rhythms remain to be investigated. Another important issue is related to the varying effects of NOB according to the clock functional state. There seems to be a general inverse correlation between NOB efficacy and clock health. For example, in young and healthy mice under normal husbandry conditions, NOB showed essentially no effects on circadian and metabolic functions, contrary to the profound improvements in obese or diabetic mouse models. ^172^^,^^178^^,^^179^ Likewise, as mentioned above, aged mice further challenged with HFD known to dampen circadian rhythms showed a greater responsiveness to NOB in healthy aging compared with aged mice fed with normal diets. ^175^ A similar pattern was observed between WT and AD mice at old ages (>22 months) where NOB was found to mitigate neuroinflammation more markedly in the latter, correlating with a more severe circadian disruption in AD mice. ^183^ These *in vivo* results together suggest a role of NOB to enhance circadian resilience toward restoring normal circadian rhythms that may have evolved to operate within a physiological range. Either dampening or indiscriminately enhancing the normal circadian rhythms is likely detrimental to organismal health.

Further research should investigate the downstream cellular mechanisms intersecting with the clock machinery. In a recent study, an inhibitory function of NOB against triple-negative breast cancer (TNBC) was found after cell line screening. ^185^ Both *in vitro* and in xenografts, NOB was able to blunt TNBC cell growth, either alone or in combination with chemotherapeutic agents. The cellular mechanism entailed, at least in part, suppression of NF-κB signaling, *via* a pathway where activation of RORs by NOB increased expression of its downstream target gene encoding IκBα, and ChIP analysis showed that ROR recruitment to the IκBα gene promoter was potentiated. ^185^ While this study illustrates a cellular pathway targeted by NOB in TNBC, it should be noted that the TNBC cells examined do not have a functional clock despite detectable clock gene expression, and NOB was not able to restore the core oscillator in these cells. ^185^^,^^186^ Therefore, this is a scenario that NOB effects are mediated by ROR transcriptional regulation independent of oscillator function. However, since the host mice have circadian rhythms, whether NOB modulates host rhythms as part of the effect against TNBC remains to be investigated. Finally, as a natural compound with an excellent safety profile, NOB is ideally suited for future trials in clinically relevant settings against clock-related disorders.

## Concluding remarks

Accumulating evidence from molecular, genetic and interventional studies highlight a critical role of the circadian secondary/stabilization loop, specifically the REV-ERBα/β and RORα/β/γ nuclear receptors, in linking the core oscillator with physiology and behavior under both normal and pathological conditions. These are multi-functional transcription factors, playing important regulatory roles in circadian regulation as well as other processes not primarily tied to the clock ( *e.g.*, RORγt in Th17 differentiation and autoimmunity). It is therefore a challenge to dissect the underlying mechanisms and devise disease-specific interventions from the circadian perspective. ^148^ Whenever possible, detailed circadian characterization should be performed, especially at the tissue and organismal levels. As illustrated by pharmacological studies targeting these factors, ^53^^,^^187^ including those on NOB, the concept of circadian resilience, or restoration of homeostatic clock function, should be an important consideration regarding intervention. Finally, given the tissue-specific nature of circadian regulation and the growing evidence for inter-organ communication with the clock system, ^188^ the functional effects, mechanistic pathways and interventional approaches should be interrogated accordingly in an integrative manner. In that regard, distribution and functional redundancy among the subtypes of these receptors should be considered. Despite the inherent complexity and practical challenges, targeting the circadian machinery, including the secondary loop, represents an exciting frontier in the 4 ^th^ dimension for research and medicine.

## Author contributions

Conceptualization: Z.C.; Original draft preparation: E.K. and Z.C.; Review and Editing: S.-H.Y. and Z.C.

## Data Availability

No data are associated with this article.
