# Supplementary material for: Circadian stabilization loop: the regulatory hub and therapeutic target promoting circadian resilience and physiological health
Source: F1000Res. 2022 Dec 12;11:1236. Originally published 2022 Oct 31. [Version 2] doi: 10.12688/f1000research.126364.2 (PMC9652504; doi:10.12688/f1000research.126364.2)
Supplement: Peer review history

# Peer review: Reviewer response for version 2

**DOI:** 10.5256/f1000research.141657.r157995

**Published:** 2022-12-13

**Authors:** Tsuyoshi Hirota

**Affiliations:** 1 Nagoya University, Nagoya, Japan

The authors addressed all my points in the revised manuscript, and I have no further comments.

Is the review written in accessible language?

Yes

Are all factual statements correct and adequately supported by citations?

Yes

Are the conclusions drawn appropriate in the context of the current research literature?

Yes

Is the topic of the review discussed comprehensively in the context of the current literature?

Yes

Reviewer Expertise:

circadian biology, small molecules

I confirm that I have read this submission and believe that I have an appropriate level of expertise to confirm that it is of an acceptable scientific standard.

# Peer review: Reviewer response for version 1

**DOI:** 10.5256/f1000research.138767.r154584

**Published:** 2022-11-11

**Authors:** Tsuyoshi Hirota

**Affiliations:** 1 Nagoya University, Nagoya, Japan

In this manuscript, Kim, Yoo, and Chen beautifully and convincingly describe the relevance of REV-ERB and ROR nuclear hormone receptors as therapeutic targets of metabolic disorders, immune diseases, brain diseases, muscle pathologies, and cancer, as well as current challenges in the research, with special emphasis on natural compound nobiletin. This well-written review article covers the translation of the molecular clock mechanism for health and healthy aging, which is an important topic in the field. I strongly support the publication of this article, and a brief description of the following points would be useful for further understanding:

- Are the therapeutic effects of nobiletin dependent on both RORα and RORγ? As the authors described, isoforms of REV-ERB (α and β) and ROR (α, β, and γ) have different expression patterns and physiological functions. Therefore, it would be nice to discuss the possibility and potential of isoform-selective ligands as well.
- Because nobiletin is a natural compound and other REV-ERB/ROR ligands are synthetic compounds, it would be nice to mention the merit (and demerit) of natural compounds compared to synthetic compounds.

Minor points:

- Page 4, line 2: Drosophila to be italic.
- Page 5, “Therapeutic relevance of REV-ERBs and RORs” section, lines 2-3: “expression” is duplicated.
- Page 6, line 11: ZT may need an explanation.
- Page 6, the second paragraph, line 1: Rev-erba-/- to be italic.
- Please check whether references 60 (Emery and Clayton, 2001) and 137 (Nagoshi et al., 2004) are proper literature in the context.

Is the review written in accessible language?

Yes

Are all factual statements correct and adequately supported by citations?

Yes

Are the conclusions drawn appropriate in the context of the current research literature?

Yes

Is the topic of the review discussed comprehensively in the context of the current literature?

Yes

Reviewer Expertise:

circadian biology, small molecules

I confirm that I have read this submission and believe that I have an appropriate level of expertise to confirm that it is of an acceptable scientific standard.

# Response

**Published:** 2022-11-28

**Authors:** Eunju Kim

**Point-by-Point Response**

 We thank both expert reviewers for their thorough and thoughtful comments. Our response is as follows.

**Reviewer 2**

**In this manuscript, Kim, Yoo, and Chen beautifully and convincingly describe the relevance of REV-ERB and ROR nuclear hormone receptors as therapeutic targets of metabolic disorders, immune diseases, brain diseases, muscle pathologies, and cancer, as well as current challenges in the research, with special emphasis on natural compound nobiletin. This well-written review article covers the translation of the molecular clock mechanism for health and healthy aging, which is an important topic in the field. I strongly support the publication of this article, and a brief description of the following points would be useful for further understanding:**

 Thank you very much for the positive comments.

**Are the therapeutic effects of nobiletin dependent on both RORα and RORγ? As the authors described, isoforms of REV-ERB (α and β) and ROR (α, β, and γ) have different expression patterns and physiological functions. Therefore, it would be nice to discuss the possibility and potential of isoform-selective ligands as well.**

 Thank you for this valuable comment. NOB shows robust binding to the LBDs of RORα and RORγ, with somewhat higher affinity for RORγ (PMID: 27076076). However, there is currently no functional evidence for a possible selectivity of NOB toward either ROR. We agree that potential isoform/subtype-selective ligands, such as those characterized for CRYs, would be valuable. We have added this discussion to the text on page 9.

**Because nobiletin is a natural compound and other REV-ERB/ROR ligands are synthetic compounds, it would be nice to mention the merit (and demerit) of natural compounds compared to synthetic compounds.**

 Thank you for this excellent comment. NOB’s excellent safety profile is indeed a significant advantage over other synthetic ligands which may require extensive medicinal chemistry efforts before in vivo and clinical applications. Without making a direct comparison, we have added a sentence on page 10 to highlight this point. Thank you.

 Minor points:

**Page 4, line 2: *Drosophila* to be italic.**

 As suggested, we have italicized the word.

**Page 5, “Therapeutic relevance of REV-ERBs and RORs” section, lines 2-3: “expression” is duplicated.**

 As suggested, we corrected the sentence.

**Page 6, line 11: ZT may need an explanation.**

 Apologies for this omission. We added the full name and explanation of ZT in the manuscript.

**Page 6, the second paragraph, line 1: *Rev-erba-/- *to be italic.**

 As suggested, we have italicized the word.

**Please check whether references 60 (Emery and Clayton, 2001) and 137 (Nagoshi *et al*., 2004) are proper literature in the context.**

 As suggested, we have removed these references as they are not immediately relevant as the reviewer pointed out.

 Thank you again.

# Peer review: Reviewer response for version 1

**DOI:** 10.5256/f1000research.138767.r154583

**Published:** 2022-11-11

**Authors:** Shihoko Kojima, Evan S. Littleton

**Affiliations:** 1 Department of Biological Sciences, Fralin Life Sciences Institute, Virginia Tech, Blacksburg, VA, USA

Summary:

 In the review article titled “Circadian stabilization loop: the regulatory hub and therapeutic target promoting circadian resilience and physiological health”, the authors discuss various studies on the role of *Rev-erbs* and *Rors* on health and aging with the main focus being their function within the circadian clock. The authors give a well-balanced and comprehensive approach to discussing the importance of these genes in many different physiological pathways, such as immune response, metabolic disorders, cancer, and more. The authors provide extensive citations to support their discussion as well. We only have minor comments, and believe the article is suitable for approval.

 Minor Concerns:

1. In the second paragraph of the section titled “The circadian timing system and health implications”, Dbp and Nfil3 are not mentioned as part of the circadian clock, despite their known regulation of multiple clock genes via D-boxes.
2. In the third paragraph of the section titled “The stabilization loop”, the authors state “While *Ror* expressions display relatively moderate circadian oscillatory amplitude, *Rev-erbs* are among the most oscillatory genes (highest amplitude) in both protein and mRNA expression”. However, *Rors* themselves differ in amplitude with *Rora* expression showing little to no rhythmicity in most tissues. Meanwhile, *Rorc *expression is rhythmic and displays similar amplitude to Rev-erbs in some tissues/cell types ^1^^,^^2^^,^^3^.
3. The nomenclature for mouse models should be superscripted (for example, *Rora ^sg/sg^* rather than *Rorasg/sg*)

Is the review written in accessible language?

Yes

Are all factual statements correct and adequately supported by citations?

Yes

Are the conclusions drawn appropriate in the context of the current research literature?

Yes

Is the topic of the review discussed comprehensively in the context of the current literature?

Yes

Reviewer Expertise:

Circadian genomics, rhythmic gene expression, mouse, clock-controlled genes

We confirm that we have read this submission and believe that we have an appropriate level of expertise to confirm that it is of an acceptable scientific standard.

## References

[1] : RORγ directly regulates the circadian expression of clock genes and downstream targets in vivo. *Nucleic Acids Res* .2012;40(17) : 10.1093/nar/gks630 8519-35 10.1093/nar/gks630 22753030PMC3458568

[2] : Genome-wide correlation analysis to identify amplitude regulators of circadian transcriptome output. *Scientific Reports* .2020;10(1) : 10.1038/s41598-020-78851-9 10.1038/s41598-020-78851-9 33318596PMC7736363

[3] : REV-ERBα and REV-ERBβ function as key factors regulating Mammalian Circadian Output. *Scientific Reports* .2019;9(1) : 10.1038/s41598-019-46656-0 10.1038/s41598-019-46656-0 31308426PMC6629614

# Response

**Published:** 2022-11-28

**Authors:** Eunju Kim

**Point-by-Point Response**

 We thank both expert reviewers for their thorough and thoughtful comments. Our response is as follows.

**Reviewer 1**

**In the review article titled “Circadian stabilization loop: the regulatory hub and therapeutic target promoting circadian resilience and physiological health”, the authors discuss various studies on the role of Rev-erbs and Rors on health and aging with the main focus being their function within the circadian clock. The authors give a well-balanced and comprehensive approach to discussing the importance of these genes in many different physiological pathways, such as immune response, metabolic disorders, cancer, and more. The authors provide extensive citations to support their discussion as well. We only have minor comments, and believe the article is suitable for approval.**

 We thank the reviewer for the overall positive comments.

 Minor Concerns:

**1. In the second paragraph of the section titled “The circadian timing system and health implications”, Dbp and Nfil3 are not mentioned as part of the circadian clock, despite their known regulation of multiple clock genes via D-boxes.**

 Apologies for this omission. We have added Dbp and Nfil3 as additional core clock genes functioning in the oscillator on page 3.

**2. In the third paragraph of the section titled “The stabilization loop”, the authors state “While Ror expressions display relatively moderate circadian oscillatory amplitude, Rev-erbs are among the most oscillatory genes (highest amplitude) in both protein and mRNA expression”. However, Rors themselves differ in amplitude with Rora expression showing little to no rhythmicity in most tissues. Meanwhile, Rorc expression is rhythmic and displays a similar amplitude to Rev-erbs in some tissues/cell types ^1,2,3^.**

 Thank you for this great comment. We have added a discussion text with these references to highlight the differences between the RORs (page 5, paragraph 2 in “the stabilization loop”). We thank the reviewer for this excellent suggestion.

**3. The nomenclature for mouse models should be superscripted (for example, Rora ^sg/sg ^rather than Rorasg/sg)**

 As suggested, we have superscripted the mouse model names.

 Thank you again.
